# Ties That Bind: Maternal and Child Health and Chronic Disease Prevention at the Centers for Disease Control and Prevention

**Published:** 2008-12-15

**Authors:** Janet L. Collins, John Lehnherr, Samuel F. Posner, Kathleen E. Toomey

**Affiliations:** Centers for Disease Control and Prevention, National Center for Chronic Disease Prevention and Health Promotion, Office of the Director; Centers for Disease Control and Prevention, Atlanta, Georgia; Centers for Disease Control and Prevention, Atlanta, Georgia; Centers for Disease Control and Prevention, Atlanta, Georgia

## Introduction

As the National Center for Chronic Disease Prevention and Health Promotion (NCCDPHP), Centers for Disease Control and Prevention (CDC), celebrates its 20th anniversary, we highlight the critical intersection of maternal and child health and chronic disease prevention. Twenty years ago, as luck — or prescient leadership — would have it, the Division of Reproductive Health (DRH) became part of the newly formed chronic disease center. Leaders inside and outside CDC have occasionally puzzled over why reproductive health work was co-located with topics such as tobacco control, diabetes, cancer, and nutrition.

Today, the logic behind this organizational decision is clear. Issues of maternal and child health, for which DRH is CDC's most visible leader, are recognized as being inextricably linked to the prevention and control of chronic disease. At the most basic level, the link is forged during pregnancy and the postpartum period, when health care providers have the opportunity to screen and treat mothers for chronic diseases, such as diabetes, and to counsel mothers on associated risk factors, such as poor nutrition and smoking (1-3). However, links between DRH and chronic disease prevention extend well beyond these obvious connections. On the one hand, the work of DRH brings heightened awareness to the importance of early intervention and its implications for lifelong health. On the other hand, expertise from diverse fields, such as tobacco control, nutrition, and diabetes, is needed to adequately address the issues of maternal, infant, and family health embraced by DRH.

## Maternal and Child Health

Consistent with the field, DRH adopts a broad definition of maternal and child health to encompass the health of women, children, and families across the life course. This approach embraces the principles of health equity, cultural competence, community empowerment, and social determinants of health in a family-centered, intergenerational approach to maternal and child health. CDC endorses the tenets of maternal and child health through clearly defined, agencywide health protection goals that support healthy people in healthy places throughout the life course. CDC goals, in turn, reside within a broader conceptual framework of "Healthiest Nations," a joint effort of the National Association of County and City Health Officials (NACCHO), the Association of State and Territorial Health Officials (ASTHO), and CDC to activate a grassroots social movement to promote health.

Given the broad focus of the field, it is not surprising that maternal and child health is an essential building block for preventing chronic disease. In fact, human epidemiologic and animal studies are beginning to show that chronic conditions may have their antecedents in compromised fetal and early postnatal periods. More established lines of research confirm that maternal health before conception, at the time of conception, and throughout pregnancy influence not only birth outcomes but morbidity as children move into adulthood (4). And the same behavioral health patterns that affect the well-being of infants and children have an even more immediate effect on the health of the mother.

Although work on the developmental origins of chronic disease is cutting-edge, we have known for years that behaviors established during childhood are critical for lifelong health. Many chronic diseases are established much earlier than previously thought. For example, obese children aged 5 to 8 years already have an average of 2 or more cardiovascular disease markers, such as high blood pressure or high cholesterol (5,6). In addition to early disease processes, obesity predisposes children to the most severe forms of obesity in adulthood; nearly 40% of obese children become morbidly obese as adults (7).

As we move from childhood into early adulthood, too many women of childbearing age already suffer from chronic conditions or use substances that can adversely affect pregnancy outcomes, leading to miscarriage, infant death, birth defects, or other complications for mothers and infants. Approximately 7% of adult women aged 18 to 44 years have asthma, 36% are overweight or obese before pregnancy, 13% are underweight, 22% use tobacco, 3% are hypertensive, 2% have diabetes, and 15% report feelings of depression during the postpartum period, according to data from the 2005 Pregnancy Risk Assessment Monitoring System (PRAMS) (written communication, Leslie Harrison, MPH, Centers for Disease Control and Prevention, November 2008). The need to intervene early in the lives of women, for their own health and that of their babies, can best be met through the joint efforts of maternal and child health and chronic disease prevention and health promotion.

## Division of Reproductive Health

To better understand pregnancy complications and maternal death and to decrease health disparities, DRH supports national and state-based surveillance systems to monitor trends and investigate health issues; conducts epidemiologic, behavioral, demographic, and health services research; and works with partners to translate research findings into health care practice, public health policy, and health promotion strategies. Through PRAMS, DRH ensures the availability of routine, high-quality state- and population-based data on women's attitudes, experiences, and behaviors before, during, and after pregnancy. Ongoing PRAMS activities and research seek answers to questions about differences in health status, risks, and outcomes related to race, ethnicity, age, geography, and other demographic factors (http://www.cdc.gov/prams/).

By leveraging partnerships, DRH's Safe Motherhood program addresses issues related to infertility, intimate partner violence, maternal complications of pregnancy, maternal depression, preconception health, prevention of HIV transmission among discordant couples, sudden unexpected infant deaths, and teenage pregnancy prevention. The division works to promote healthy pregnancy outcomes and to better understand how to address disparities. DRH is accomplishing its work by building partnerships to address urgent issues such as preterm birth, the most frequent cause of infant death in the United States (http://www.cdc.gov/ReproductiveHealth/).

DRH's work extends beyond US borders by providing support and technical assistance and by building capacity for improving infant health, optimizing maternal health, enhancing women's reproductive health, and helping prevent unintended pregnancy, particularly in developing countries. The October 2008 issue of *Preventing Chronic Disease* highlighted DRH's work on the US-Mexico border. With the impending assignment by DRH of a senior maternal and child health epidemiologist, Jill McDonald, PhD, to the US-Mexico Border Health Commission, we eagerly anticipate the expanded use of data to improve maternal and infant health outcomes on both sides of the border (8,9).

DRH has a long history of working with national and international partner organizations to reduce mortality and morbidity in conflict and disaster situations. One example of DRH's contributions in this area is the Reproductive Health Assessment Toolkit for Conflict-Affected Women, which contains tools to assess the reproductive health needs of women and their families in conflict areas and guidance on how to use relevant data to promote and enhance programs and services (http://www.cdc.gov/ReproductiveHealth/Refugee/index.htm).

DRH's work fits into a broad professional landscape, with colleagues in the Health Resources and Services Administration's Maternal and Child Health Bureau, ASTHO and its affiliates, NACCHO, the US Agency for International Development (USAID), the World Health Organization, the United Nations Children's Fund, the World Bank, the Association of Maternal and Child Health Programs (AMCHP), and CityMatCH. CDC resources have enabled AMCHP to provide much-needed training opportunities for maternal and child health epidemiologists working at the state and local levels (http://www.amchp.org/Pages/Welcome.aspx and http://www.amchp.org/publications/WomensHealth/Documents/ChronicFactSheet.pdf). CityMatCH, which is the leading source for maternal and child health data and analytic tools in urban areas of the United States, is coordinating several DRH-supported initiatives, including advancing preconception care, conceptualizing and implementing the life-course perspective, and reducing racial and ethnic disparities in infant health (http://www.citymatch.org/). DRH and the Council of State and Territorial Epidemiologists jointly sponsor a fellowship program that trains recent graduates in the expanding field of applied maternal and child health epidemiology. This 2-year fellowship provides a high-quality training experience under the guidance of an experienced mentor and secures long-term career placements for fellows at the state or local level (http://www.cste.org/dnn/). Internationally, DRH contributes to the scientific foundation on which USAID Washington and Missions, other donors, and host-country institutions make evidence-based decisions to improve reproductive health worldwide.

A recent focus for DRH's adolescent reproductive health team is the promotion of youth assets (eg, strong parent-child communication, connectedness to school, belief in the future) based on a youth development paradigm. This paradigm highlights the links between youth assets and educational success, social and emotional competence, and reduced risk behaviors, such as those leading to teen pregnancy (10-12). These DRH efforts complement the work of NCCDPHP's Division of Adolescent and School Health, which supports schools, parents, and communities in promoting the health and safety of young people. One such activity is the division's partnership with DRH to encourage schools and community-based, youth-serving organizations to adopt science-based approaches to prevent teen pregnancy and sexually transmitted infections, including HIV.

## National Center for Chronic Disease Prevention and Health Promotion

Surrounding DRH in NCCDPHP are 9 divisions and multiple units that support maternal and child health through their work in school health, oral health, genomics, tobacco control, alcohol use, heart disease, stroke, cancer, diabetes, nutrition, physical activity, and obesity. For example, groundbreaking maternal and child health-related work can be found in the Division of Adult and Community Health, which conducts the Adverse Childhood Experiences (ACE) study in collaboration with Kaiser Permanente. The ACE study reveals that abuse, neglect, and exposure to other traumatic stressors during early childhood profoundly influence health and behavioral outcomes later in life (13) (http://www.cdc.gov/nccdphp/ace/).

The work of the Division of Nutrition, Physical Activity, and Obesity (DNPAO) is at the heart of maternal and child health. DNPAO leads CDC's efforts to promote breastfeeding as a way to reduce infections, asthma, and overweight among infants and children, as well as to help mothers attain normal weight and reduce their risk of some cancers. DNPAO supports research about breastfeeding and shares practical strategies, policies, and tools to promote the practice in the United States and around the world (http://www.cdc.gov/nccdphp/dnpa/). DNPAO also conducts the Pediatric Nutrition Surveillance System and the Pregnancy Surveillance System to monitor the nutritional status of low-income infants, children, and women in federally funded maternal and child health programs. These surveillance systems provide data that describe prevalence and trends of nutrition, health, and behavioral indicators for mothers and children.

DRH also works closely with its colleagues in NCCDPHP's Office on Smoking and Health in preventing tobacco use before, during, and after pregnancy. According to 2005 PRAMS data from 16 states, 22% of women smoked cigarettes 3 months before pregnancy, 14% smoked during pregnancy, and 16% smoked after delivery (measured at an average of 4 months). Women who quit smoking before or early in pregnancy significantly reduce their risk, and that of their baby, for several adverse health outcomes (14,15). As the lead federal agency for tobacco use prevention, the Office on Smoking and Health also works to reduce exposure to secondhand smoke, particularly among infants and children, whose developing bodies are especially vulnerable to the poisons in secondhand smoke (16). Its Internet-based Maternal and Child Health Smoking-Attributable Mortality, Morbidity, and Economic Cost program allows users to estimate the health and health-related economic consequences of smoking on neonatal outcomes. State maternal and child health and tobacco control programs use these data for education, advocacy, and policy efforts (http://www.cdc.gov/Reproductivehealth/PrenatalSmkbk/data_sources_definitions.htm#MCH).

The center's Division of Diabetes Translation provides public health leadership to prevent the onset of diabetes, promote early diagnosis, and improve treatment and outcomes for people with diabetes. Diabetes that occurs either before pregnancy or during pregnancy (gestational diabetes) is associated with negative health outcomes for mothers and babies, such as birth defects, miscarriage, and heart and kidney disease (http://www.cdc.gov/Features/DiabetesPregnancy/). The division helps women — especially those at greatest risk for health disparities — better manage the disease and promotes policies that improve quality of and access to care (http://www.cdc.gov/diabetes/index.htm). Approximately 4% of pregnancies are affected by gestational diabetes. Women with gestational diabetes should be screened for diabetes following pregnancy, because they are at higher risk for developing type 2 diabetes later in life (2,3). The division, in collaboration with the American College of Obstetricians and Gynecologists and DRH, is developing an educational toolkit for obstetric providers and for patients with gestational diabetes.

More than 80% of women of childbearing age have dental caries or other oral diseases that are associated with complications for women and infants (17). A study by NCCDPHP's Division of Oral Health found that most mothers did not visit a dentist during pregnancy, and of those who reported having oral health problems, half did not seek care (18). The division is working to increase the attention given to the oral health needs of pregnant women (http://www.cdc.gov/OralHealth/).

The work of the Office of Public Health Genomics is also key to the success of maternal and child health research and programs. The completion of the Human Genome Project and advances in genomic technologies have ushered in a new era of personalized health and targeted public health interventions. The office supports public health research activities related to preterm delivery and other maternal and child health outcomes, develops approaches to the use of family history to improve health and prevent disease, evaluates the use of genetic testing and screening (eg, newborn screening) to improve population health, and develops public health capacity and competencies to integrate new genomic tools into practice (http://www.cdc.gov/genomics/).

The brevity of this editorial prevents us from describing all of the ways that DRH interacts with its chronic disease division counterparts. Work in the Division of Cancer Prevention and Control on cancer survivorship and infertility, in the Division of Adult and Community Health on alcohol use, and in the Division for Heart Disease and Stroke Prevention on congenital heart disease and other cardiovascular health threats have all been essential to our maternal and child health efforts.

Despite these omissions, we hope we have succeeded in showcasing the increasingly interdependent work of DRH and other divisions within NCCDPHP during the past 20 years.  Contributions to maternal and child health can be seen in nearly every division in the center, and it is easy to appreciate the vital contributions of DRH to the prevention and control of chronic disease. Although the efforts of DRH and its sister NCCDPHP divisions are closely intertwined, together they reflect only a portion of CDC's investment in maternal and child health. Nearly every center at CDC contributes to maternal and child health through pivotal work in areas such as birth defects and developmental disabilities, injury, violence against women, immunization, HIV/AIDS and other sexually transmitted diseases, environmental health, infectious diseases, and preparedness.

In 2004, NCCDPHP joined the National Center for Birth Defects and Developmental Disabilities (NCBDDD) as part of CDC's newly formed Coordinating Center for Health Promotion. This organizational pairing has resulted in dramatically increased partnerships. A notable example is DRH and NCBDDD's joint work with partners to identify 10 key recommendations to improve preconception health (http://www.cdc.gov/ncbddd/preconception/). These recommendations promote optimal health throughout the life course for women, children, and families by focusing on high-priority clinical care and population-based public health strategies.

The extent of maternal and child health work at CDC is encouraging and exciting. Unfortunately, navigating such a complex array of resources can be daunting, if not impossible. This brief overview of the breadth of maternal and child health work, even within the confines of just 1 center, leads us to conclude that the field might benefit from a virtual maternal and child health community at CDC. In its leadership role on maternal and child health, DRH plans to make this virtual community a reality by building from the existing pregnancy gateway (http://www.cdc.gov/ncbddd/pregnancy_gateway/) and expanding it to provide a "one-stop shopping" maternal and child health Web portal at CDC.

As we celebrate NCCDPHP's 20th anniversary, we are reminded that DRH has delivered more than 40 years of public health leadership at CDC. We commend its staff for their extraordinary contributions and look forward to building even stronger bonds between reproductive health and chronic disease prevention in the decades to come.
